# Sarcocystosis of chital-dhole: conditions for evolutionary stability of a predator parasite mutualism

**DOI:** 10.1186/1472-6785-5-3

**Published:** 2005-02-22

**Authors:** Maithili M Jog, Milind G Watve

**Affiliations:** 1 Department of Microbiology, Abasaheb Garware College, Karve Road, Pune 411 004, India; 2 Life Research Foundation 10, Pranav, 1000/6-c Navi Peth, Pune 411 030, India

## Abstract

**Background:**

For parasites with a predator-prey life cycle, the completion of the life cycle often depends on consumption of parasitized prey by the predator. In the case of such parasite species the predator and the parasite have common interests and therefore a mutualistic relationship is possible. Some evidence of a predator-parasite mutualism was reported from spotted deer or chital (*Axix axis*) as a prey species, dhole or Indian wild-dog (*Cuon alpinus*) as the predator and a protozoan (*Sarcocystis axicuonis*) as the parasite. We examine here, with the help of a model, the ecological conditions necessary for the evolution and stability of such a mutualistic relationship. A two – level game theory model was designed in which the payoff of a parasite is decided not only by alternative parasite strategies but also by alternative host strategies and vice versa. Conditions for ESS were examined.

**Results:**

A tolerant predator strategy and a low or moderately virulent parasite strategy which together constitute mutualism are stable only at a high frequency of recycling of parasite and a substantial prey – capture benefit to the predator. Unlike the preliminary expectation, parasite will not evolve towards reduced virulence, but reach an optimum moderate level of virulence.

**Conclusion:**

The available data on the behavioral ecology of dhole and chital suggest that they are likely to meet the stability criteria and therefore a predator-parasite mutualism can be stable in this system. The model also points out the gaps in the current data and could help directing further empirical work.

## Background

Preferential killing of sick and disabled prey individuals by the predator has been the focus of many ecologists working with different predator – prey systems. In a variety of prey predator systems, diseased or weaker animals are shown to be consumed in greater proportion by predators [1-5]. Increased susceptibility of parasitized prey to predation, or predator preference for parasitized prey is possible under a set of conditions [6-8]. Where the prey species is an intermediate host and the predator is the definitive host for a parasite species, the capture of prey is often an essential part of the life cycle. Therefore any mechanism that makes the prey susceptible to predation would enhance the parasite fitness. In such relationships the susceptibility induced by the parasite can be very specific towards the predator host [9]. A mutualistic relationship can be said to exist between a predator and a parasite [10] if the cost of harboring the parasite is less than the benefit of greater success in catching the prey [1]. Some evidence suggestive of predator-parasite mutualism comes from dhole or Indian wild dog (*Cuon alpinus*) and a protozoan parasite (*Sarcosystis axicuonis*) with chital or spotted deer (*Axis axis*) as the prey-host [1,11].

There can be a potential problem in such a mutualistic relationship. Low virulence of the parasite towards the predator host and parasite tolerance by the predator host are essential factors for the maintenance of a mutualistic relationship. However, it is possible that a virulent parasite can grow faster and invade a mild parasite population. On the other hand a parasite resistant predator can avoid the cost of parasitism but share the benefit of prey capture and therefore invade a tolerant population. Either of the events can destabilize the mutualistic relationship. It is essential therefore to examine the evolutionary stability of the mutualism. In a completely randomized distribution, a mild parasite population can be easily invaded by a virulent one and a tolerant predator can be invaded by a resistant one. Population viscosity, group selection and kin selection can alter the evolution of virulence [12]. The dhole – *Sarcocystis *system makes group selection and viscosity very likely factors in shaping the relationship [1]. The life cycle of the parasite is very short as compared to dhole life span. Dhole groups are stable and retain their territories over a long time. Dhole territories are large and encompass home ranges of several chital packs [13,14]. Therefore the protozoan harboured by a dhole pack is very likely to be recycled to the same pack. The benefit of the parasite is more likely to be gained by the same pack. The distribution of a parasite within a pack is shown to follow a consistent pattern in which only a few individuals carry most of the parasite load. On the other hand, one or two individuals in each pack are found to be parasite free. This suggests that within a pack there can be distribution of labor. [1]. A distribution of labor, in which some individuals do active hunting and some disseminate the parasite, can reduce the effective cost of carrying the parasite. Evolution can take a different route under such conditions.

We examine here the effect of parasite recycling on the evolution of a predator-parasite mutualism, using a theoretical model.

### The model

We consider two alternative strategies, namely mild and virulent, for the parasite (Table 1). The virulent parasite multiplies rapidly in the predator host and therefore enjoys greater success (v) and exerts a higher cost (x) on the predator host. The mild parasite exerts relatively low cost (y) on the predator host and gains a limited success (m). The predator has two alternative strategies, namely tolerant and resistant. A tolerant predator always harbors the parasite population whereas the resistant one attempts to resist or eliminate the parasite. However, since the parasite virulence mechanisms also evolve, there is a probability (p) that the parasite can infect a resistant predator. The predator gets an additional net benefit (z) from capturing a prey infected with the parasite as compared to capturing an uninfected prey. The prey can have only one viable strategy, that of becoming resistant to the parasite. The prey will not get any benefit by tolerating the parasite since it would make it more susceptible for predation. Therefore, we do not consider alternative prey strategies in the model.

**Table 1:** Pay – off matrix for predator and parasite strategies.

		parasite		predator	
	
		Mild	Virulent	Tolerant	Resistant
	
parasite	Mild	1-y	fr(1-y)+(1-fr)(1-x)	m	p* m
	Virulent	fr(1-x)+(1-fr)(1-y)	(1-x)	v	p* v
predator	Tolerant	-y	-x	z	(1-fr) p *z + fr *z
	Resistant	- p * y	- p* x	(1-fr)*z + fr (p* z)	p* z

The table differs from pay-off matrix tables for classical game theory models. The table accounts for two alternative strategies each for two different types of players namely parasite and predator. The pay-off of the parasite is not only decided by other parasites but also by the predator strategy and vice-versa. Therefore the complete pay-off of a mild parasite invading a virulent population in a tolerant host population is m * [fr(1-y)+(1-fr)(1-x)]. Others to be calculated similarly.

If the parasite population consists of the mild type, they enjoy a fitness of 'm' from the tolerant host and 'p*m' from a resistant one. Since they exert a cost y on the host, there is erosion of the host resource. The host resource available to them is therefore (1-y). Similarly for a population of virulent parasites the mean fitness gain is v and the host resource available (1-x). A virulent host invading a mild population will gain a fitness of 'v' such that v > m. In the absence of recycling the host resource available to it would be (1-y). However with a frequency of recycling 'fr', the host resource would be,

fr (1-x) + (1-fr)(1-y)

Similarly, that for a mild parasite invading a virulent population would be,

fr (1-y) + (1-fr)(1-x)

If the predator population is tolerant the parasite will be harbored in large numbers and disseminated to the prey population. Since the parasitized prey is more susceptible to predation the predator gets a benefit 'z' of easy catching. A resistant population, on the other hand has a small probability 'p' of harboring the parasite. Therefore the benefit the predator gets would be 'p*z'. If a resistant predator invades a tolerant population, with the recycling factor 'fr', the benefit of prey capture would be,

fr * p *z + z(1-fr).

The benefit for a tolerant one invading a resistant population would be,

fr * z + (1-fr)* p*z.

We assume that 'v', the benefit for a virulent parasite by infecting single host is directly proportional to 'x' i.e the loss to the host from infection by virulent parasite.

V = α *x similarly, m = α *y

## Results and discussion

A mild parasite will be able to invade a virulent population if the pay-off to the mild invader is greater than that for the virulent population. When the predator population is tolerant, this condition is satisfied when,

y * [fr (1-y)+(1-fr) (1-x)] > x (1-x)

The condition under which a virulent invader is unable to invade a mild population is

(1-y)* y > [fr *(1-x) + (1-fr)*(1-y)]*x

Since x > y, for satisfying both these conditions, fr should be large, y should be moderate and x should be large. Thus selection would favour a moderate virulence in the parasite towards the predator host. Unlike our expectation, low virulence is unlikely to be stable. However, a mutualistic relation can remain if the prey capture benefit is sufficiently large. It can be easily seen that the above conditions remain unaffected even if the predator population is resistant.

Considering predator strategies, a tolerant predator will be able to invade a resistant population in the presence of a mild predator if,

p *z - p*y < (1-fr) p *z + fr*z - y

i. e. p*fr * z - p* y < fr *z - y

This condition will be satisfied if fr *z > y since p < 1. A resistant predator will be unable to invade a tolerant population since the necessary condition is

z-y < (1-fr)*z + fr*(p*z) - (p * y)

fr*z - y < p*fr*z - p*y

This invasion is impossible if fr*z > y. If the parasite is virulent, the necessary condition would be fr*z > x. Since x is assumed to be large, a resistant predator would be stable if the prevalent parasite strategy is virulent.

Thus when fr and z are large and y is small mutualism would be stable. When this condition is not satisfied the predator will evolve resistance to the parasite and the parasite will evolve greater virulence.

## Conclusion

A large recycling frequency (fr) appears to be the only critical factor in the evolution of parasite virulence. However, the parasite is unlikely to evolve towards low virulence. There will be a moderate virulence optimum. For a net benefit to the host the cost associated with this level of virulence should be less than the benefit in terms of ease of prey capture.

For the evolution and stability of the tolerant strategy in the predator a large fr as well as large z and small y are necessary. A predator-parasite mutualism therefore critically depends upon these factors, whereas it is independent of p and α.

In the case of Dhole-chital – *Sarcocystis *system, these condition are very likely to be satisfied. Field data show that the frequency of sarcocystosis of the heart in dhole kills was approximately double that of chital dyeing of other causes [1]. This suggests a substantially large z. Dhole have large, stable and defended territories upto 80 Km^2 ^[13]. There is only marginal overlap between neighboring packs unlike the overlap in tigers [15]. The territory of a dhole pack encompasses the home ranges of several chital groups. The home ranges of chital groups are small and stable [13,16]. This can ensure a large 'fr'. Unlike wolves, which tend to defecate more on the boundaries of the territory [17], the frequently used defecation sites of dhole tend to be towards the center of their home range and close to the major hunting areas [18], further ensuring a large 'fr'. The intensity of intestinal infection is reflected in the density of sporocyst in Dhole scat. Dhole shading large numbers of sporocysts of *S. axicuonis *show no apparent symptoms of disease or abnormality [19,20]. This indicates that the virulence of *S. axicuonis *towards dhole is low. Further, the division of labor can substantially reduce the effective cost of carrying the parasite. Currently we are unable to quantify these parameters empirically and have no estimate of the actual cost of harboring the parasite. Therefore we are unable to state quantitatively that all the necessary conditions for mutualism are met by the system. The importance of the model is that it helps us identify the gaps in the data and thus orient future empirical work.

Although we are far from having an empirical estimate of y, fr and z, the known ecology of chital and dhole suggest that fr and z could be sufficiently large. This makes the system a likely candidate for the evolution of predator parasite mutualism. Any other predator-prey system that satisfies these conditions is also likely to co-evolve with some parasite species towards a predator-parasite mutualism. Parasites of diverse taxa have evolved predator-prey life cycles and any of them could be possible candidates for a mutualism. Predator-prey-parasite systems that satisfy the following three criteria are the most likely candidates for a stable mutualistic relationship:

i) parasitized prey individuals are killed with substantially greater frequency by the predator

ii) pathogenicity of the parasite towards the predator host is low or moderate

iii) there is a high rate of parasite recycling to the predator host.

We need to look at a number of systems that could satisfy these criteria. The chital-dhole-*Sarcocystis *system may not be unique and many possible examples of predator-parasite mutualism may be present in nature.

## Authors' contributions

Both authors have contributed approximately equally to the model development.
